# A re-evaluation of random-effects meta-analysis

**DOI:** 10.1111/j.1467-985X.2008.00552.x

**Published:** 2009-01

**Authors:** Julian P T Higgins, Simon G Thompson, David J Spiegelhalter

**Affiliations:** Medical Research Council Biostatistics UnitCambridge, UK

**Keywords:** Meta-analysis, Prediction, Random-effects models, Systematic reviews

## Abstract

Meta-analysis in the presence of unexplained heterogeneity is frequently undertaken by using a random-effects model, in which the effects underlying different studies are assumed to be drawn from a normal distribution. Here we discuss the justification and interpretation of such models, by addressing in turn the aims of estimation, prediction and hypothesis testing. A particular issue that we consider is the distinction between inference on the mean of the random-effects distribution and inference on the whole distribution. We suggest that random-effects meta-analyses as currently conducted often fail to provide the key results, and we investigate the extent to which distribution-free, classical and Bayesian approaches can provide satisfactory methods. We conclude that the Bayesian approach has the advantage of naturally allowing for full uncertainty, especially for prediction. However, it is not without problems, including computational intensity and sensitivity to *a priori* judgements. We propose a simple prediction interval for classical meta-analysis and offer extensions to standard practice of Bayesian meta-analysis, making use of an example of studies of ‘set shifting’ ability in people with eating disorders.

## 1 Introduction

In systematic reviews, which attempt to assemble the totality of evidence that is relevant to specific research questions, a typical approach to meta-analysis is to average the estimates of a comparable parameter from each study. A ‘fixed effect’ model assumes that a single parameter value is common to all studies, and a ‘random-effects’ model that parameters underlying studies follow some distribution. Occasionally it may be reasonable to assume that a common effect exists (e.g. for unflawed studies estimating the same physical constant). However, such an assumption of *homogeneity* can seldom be made for studies in the biomedical and social sciences. These studies are likely to have numerous differences, including the populations that are addressed, the exposures or interventions under investigation and the outcomes that are examined. Unless there is a genuine lack of effect underlying every study, to assume the existence of a common parameter would seem to be untenable.

If a common effect cannot be assumed then *heterogeneity* is believed to be present. The effects may be

- assumed different and unrelated
- assumed different but similar or
- modelled by using covariates.

In the first, each study is considered in isolation from the others and meta-analysis is ruled out as an option. In the second, a random-effects model may be assumed to reflect the similarity. In the third, a regression analysis is suggested. Although sources of heterogeneity should ideally be investigated (Thompson, 1994), selection of covariates to explain heterogeneity can be difficult, both practically (Higgins *et al.*, 2002) and theoretically (Thompson and Higgins, 2002). Where no covariates are obvious contenders to explain the heterogeneity, random-effects meta-analysis is appropriate and is the situation that we address primarily in this paper. Many of our arguments also apply, however, when heterogeneity may be *partially* explained by using covariates and residual heterogeneity is modelled by using a random effect.

The focus of this paper is a re-evaluation of the random-effects approach to meta-analysis in an attempt to gain a better understanding of its purpose and interpretation. We first discuss the important questions to be addressed. We then examine the extent to which classical and Bayesian methodology, both non-parametric and parametric, can satisfactorily address the important questions (Sections 3–6). An example is introduced in Section 7, and in Section 8 we address some special issues in random-effects meta-analysis, including the implications of potential bias in the estimates from component studies.

## 2 Important questions to address in a random-effects meta-analysis

We structure our discussion of random-effects meta-analysis around three basic aims of statistical methodology, namely estimation, prediction and hypothesis testing. The division is somewhat artificial, since there is some overlap across the domains. We recognize that different perspectives will be relevant for different purposes.

We represent the effect underlying the *i*th of *k* studies by *θ*_*i*_. For the most part we consider these to be drawn from some unspecified distribution *f*(Φ), with parameters Φ, such that *E*[*θ*_*i*_]=*μ* and var(*θ*_*i*_)=*τ*^2^. The assumption of a common between-study variance for each study is a strong one that we return to in Section 8.1. For generality, the situation that we address assumes only the availability of effect estimates 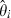 that are unbiased for *θ*_*i*_ (*i* =1,…,*k*), with sampling variance (i.e. squared standard error) 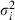. We shall follow the conventional assumption that estimates 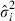 (which are independent of 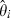) may be used in place of the true variances, and we use the two interchangeably. No specific distributional forms are assumed at this stage.

### 2.1 Estimation in random-effects meta-analysis

In practice, the prevailing inference that is made from a random-effects meta-analysis is an estimate of underlying mean effect *μ*. This may be the parameter of primary interest: for example, the average efficacy of a treatment may be the most relevant parameter for health care providers (Feinstein, 1995; Light, 1987; Ades *et al.*, 2005). Estimates of *μ* are frequently misinterpreted as ‘the overall effect’. However, a single parameter cannot adequately summarize heterogeneous effects (Raudenbush and Bryk, 1985) and so focusing on *μ* alone is usually insufficient.

**Fig. 1:**
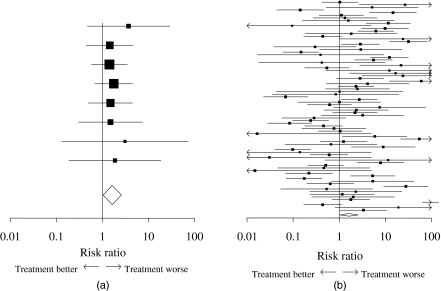
Estimates with 95% confidence intervals for (a) a genuine meta-analysis and (b) an artificially constructed meta-analysis with identical results for the mean of a random-effects distribution

To illustrate the problem consider Fig. 1, which illustrates two meta-analytic data sets. The first is a meta-analysis from a systematic review of human albumin treatment for critically ill patients (Cochrane Injuries Group Albumin Reviewers, 1998). There is no heterogeneity in log-risk-ratios among these eight trials (method-of-moments estimate 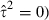, so a traditional random-effects meta-analysis coincides numerically with a meta-analysis assuming a common effect. The second data set we constructed artificially to contain 80 studies with highly heterogeneous findings. The larger number of trials decreases uncertainty of the estimated mean effect, whereas the presence of heterogeneity increases uncertainty. These counteract each other such that a random-effects analysis produces an estimate 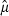 and standard error that are identical to the eight genuine trials. However, there are important differences between the two data sets that would lead to very different interpretations and consequences for clinical practice. The variation in directions and magnitudes of effect in the artificial data set would suggest that the effect is highly variable, and that some exploration of the heterogeneity is warranted.

The naive presentation of inference only on the mean of the random-effects distribution is highly misleading. Estimation of *τ*^2^ is just as important. This variance explicitly describes the extent of the heterogeneity and has a crucial role in assessing the degree of consistency of effects across studies, which is an element of random-effects meta-analysis that often receives too little attention. It is also important to be able to express uncertainty surrounding the estimate of *τ*^2^. There might also be interest in estimating the study-specific treatment effects *θ*_*i*_, or in obtaining ‘refined’ estimates of effects in one or more specified studies that most closely resemble a real life situation.

### 2.2 Prediction using random-effects meta-analysis

Predictions are one of the most important outcomes of a meta-analysis, since the purpose of reviewing research is generally to put knowledge gained into future application. Predictions also offer a convenient format for expressing the full uncertainty around inferences, since both magnitude and consistency of effects may be considered. A major benefit of a random-effects model over the common effect model is that inferences can be made for studies that are not included in the meta-analysis, say for *θ*_new_ drawn from *f*(Φ).

Realistic interpretation of predictions from a random-effects model can, however, be difficult. For instance, the predicted effect *θ*_new_ relates to the effect in a new study that is deemed ‘sufficiently similar’ to the existing studies to be eligible for the analysis; in Section 4 we provide a formal definition of ‘similar’ in terms of ‘exchangeability’. It will usually be more relevant to predict true effects (akin to the *θ*_*i*_) rather than effect estimates (the 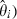. Prediction intervals for the latter depend on the sample size and other characteristics of the population (e.g. anticipated risks, or outcome standard deviations) and so may only have practical use in the design of an actual study.

### 2.3 Statistical tests in the presence of heterogeneity

An important rationale for meta-analyses is the possibility of increased power, compared with the individual studies, to detect moderate effects. Compatibility of results with a null hypothesis can be at least as useful as estimation of overall effect sizes, because in many meta-analyses the studies are too diverse to compare estimates, while being sufficiently similar in their aims to be examining comparable null hypotheses. Here we discuss possible tests in the context of meta-analysis in the presence of heterogeneity. Extensive discussions of meta-analytic tests, particularly relating to common effect analyses, appear elsewhere (Hedges and Olkin, 1985; Bailey, 1987; Thompson, 1998). We start by listing five potential null hypotheses.

A basic null hypothesis of general interest in a meta-analysis is 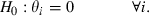
(1)

Acceptance of this hypothesis is acceptance that no genuine effect exists in any study, which is a strong but important conclusion. This is the one situation in which homogeneity of (a lack of) effect is plausible. The usual heterogeneity test examines a similar null hypothesis that all effects are equal but allows for this effect to be non-zero: 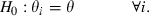


We have argued that such a null hypothesis is typically untenable and have discouraged the use of homogeneity tests in favour of quantifying the extent of heterogeneity (Higgins and Thompson, 2002). The usual null hypothesis that is associated with a random-effects analysis is whether the underlying mean effect *μ* =*E*[*θ*_*i*_] is non-zero: 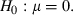
(2)

All these null hypotheses are non-directional. Meta-analyses are often built on an assumption that directions of effect are consistent across studies (Peto, 1987), yet formal tests of this important supposition are not routinely undertaken. Two null hypotheses that do address this are 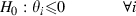
(3) and, in the context of prediction, 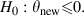
(4)

The simplest alternative to the basic null hypothesis (1) is that an effect exists in at least one study: 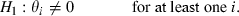
(5)

Other alternative hypotheses are feasible. The conventional test from a common effect meta-analysis is a test of hypothesis (1) that is particularly powerful against the alternative hypothesis 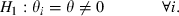


Although often useful, the test may be misleading if there is heterogeneity and in particular will not reject hypothesis (1) if effects are variable and centred on 0. This property is shared with the conventional test from a random-effects meta-analysis, i.e. of hypothesis (2) against 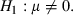


More specifically, neither test can distinguish between the null hypotheses (1) and (2). To investigate whether effects are in the same direction, we can compare hypothesis (3) with 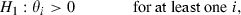 or hypothesis (4) with 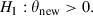


We review methods for examining these hypotheses in Section 5.

### 2.4 Summary: important objectives in random-effects meta-analysis

We propose that the following five aspects are likely to be relevant and useful results from a random-effects meta-analysis. The exact selection from these for a specific meta-analysis will depend on its aims and context. We assume *a priori* that if an effect exists then heterogeneity exists, although it may be negligible. A sole focus on estimating the mean of a random-effects distribution may be misleading, as demonstrated in Fig. 1, and emphasis should be placed on the whole distribution of underlying effects.

- *Heterogeneity*—quantification of heterogeneity of findings—the extent of inconsistency is important; a test of homogeneity is not.
- *Mean effect*—estimation of the underlying mean *μ*—this is clearly important but it provides an *incomplete summary*.
- *Study effects*—estimation of study-specific effects *θ*_*i*_—this may be of interest if studies are distinguishable from each other in ways that cannot be quantified, or in more respects than can reasonably be investigated by using a regression approach.
- *Prediction*—prediction of effect in a new study, *θ*_new_—predictive distributions are potentially the most relevant and complete statistical inferences to be drawn from random-effects meta-analyses.
- *Testing*—testing of whether an effect exists in any study, whether an effect has a consistent direction and/or whether an effect is predicted to exist in a new study—these may be more important questions than whether or not an effect exists *on average*.

We now discuss how well these objectives can be met, in turn, by assuming nothing about the distribution of random effects, by assuming a normal distribution for the random effects and by allowing a more flexible distribution for the random effects.

## 3 Meeting the important objectives with no distributional assumption for the random effects

One widely quoted criticism of random-effects meta-analysis is the need to assume a distribution for the random effects in the absence of a sound justification. Here we outline the extent to which a random-effects approach can meet our important objectives in the absence of any distributional assumption about the random effect. We are willing to assume normality of the effect estimates from each study, so
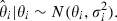
(6)

### 3.1 Heterogeneity

A point estimate of the heterogeneity variance *τ*^2^ is available by using a moment-based approach (Whitehead and Whitehead, 1991; DerSimonian and Laird, 1986): 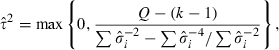 where 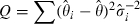
(7) and 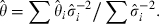


The variance of *Q*, and hence a variance for 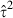, has been derived (Biggerstaff and Tweedie, 1997). For very large data sets this could form the basis of a symmetric confidence interval. Alternatively, a gamma distribution has been proposed as an approximation to the skewed distribution of *Q*, and this may form the basis of an asymmetric confidence interval for *τ*^2^ (Biggerstaff and Tweedie, 1997). Alternatively, we have previously developed functions of *τ*^2^, such as the proportion of variability in the 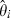 that is attributable to heterogeneity, that assess the effect of heterogeneity and may be compared across all meta-analytic situations (Higgins and Thompson, 2002).

### 3.2 Mean effect

A typical classical approach to random-effects meta-analysis (Whitehead and Whitehead, 1991; DerSimonian and Laird, 1986) takes as an estimate of *μ* the weighted average 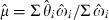, where ‘inverse variance’ weights are 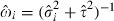. The approximate standard error of 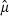 is given by 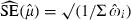. These results are not dependent on a distributional assumption for the random effects. Invoking the central limit theorem, an approximate 95% confidence interval may be obtained as 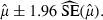
(8)

This interval will be valid approximately in a distribution-free context when there are many studies.

An alternative is a quasi-likelihood approach in which the meta-analysis is performed under a common effect assumption (*τ*^2^=0) and the standard error of the estimate inflated to account for heterogeneity (Detsky *et al.*, 1992). For example, for Poisson and logistic models the standard error may be multiplied by √{*Q*/(*k*−1)} (Tjur, 1998) which approximates the ratio of standard errors from usual random-effects analysis and common effect analyses (Higgins and Thompson, 2002).

### 3.3 Study effects

Arguments that were put forward by Stein (1956), and those who have subsequently developed empirical Bayes methods, indicate that an appropriate estimate of a study-specific effect is a weighted average of that study's observation and an average across all studies. In particular, for the case of all estimates having the same (known) sampling error (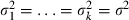, say), and with *τ*^2^ known, then the linear function of *y*_*i*_ that minimizes the expected mean-squared error is (Robbins, 1983) 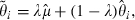 where *λ* =*σ*^2^/(*σ*^2^+*τ*^2^).

### 3.4 Prediction

Attempts to use the empirical distribution of observed effect sizes, 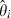, to predict future effects should be avoided. The distribution of the estimates is overdispersed, since the true variance of an individual 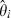 around the population mean *μ* is 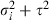, not *τ*^2^. Further, the sampling errors (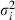
s) may be substantially different for different studies. A histogram of observed results, for example, may therefore be misleading.

Predictive distributions and prediction intervals do not follow naturally from distribution-free approaches. The central limit theorem, although leading to an approximate confidence interval for *μ*, is not relevant to a prediction interval for the effect in a new trial, *θ*_new_. However, (impractically wide) intervals would be available via Chebyshev's inequality, which states that no more than 1/*t*^2^ of the values are more than *t* standard deviations away from the mean (and, thus, at least 95% of true effects would lie within √20=4.47 standard deviations of the mean).

### 3.5 Testing

Three hypothesis tests are in common use as part of a classical meta-analysis. These are a test for non-zero effect under a common effect model, a test for non-zero mean effect under a random-effects model and a test of homogeneity of effects across studies. The first and third do not rely on distributional assumptions for the nature of any heterogeneity that may exist. The random-effects test is built on an assumption of normality for the *θ*_*i*_.

None of these tests address the questions that we consider to be important in a random-effects meta-analysis. Our initial question of whether there is evidence for some treatment effect among the studies may be addressed by using the test statistic 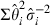. This follows a 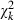-distribution under the null hypothesis (1) and provides a test against the alternative (5). Unfortunately the test has poor power, like the test of homogeneity *Q* in equation (7) (Hardy and Thompson, 1998) of which it is a special case (assigning 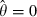 and providing an extra degree of freedom).

To address our second question, of whether all treatment effects lie in the same direction, we might make use of a test for *qualitative interaction*. Such tests have been developed (Gail and Simon, 1985; Piantadosi and Gail, 1993; Pan and Wolfe, 1997), addressing a null hypothesis: 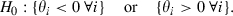


Gail and Simon (1985) described the use of a test statistic 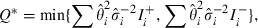
(9) where 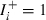 if 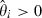 and 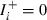 otherwise; 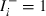 if 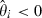 and 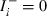 otherwise. Critical values for the statistic were given in Gail and Simon (1985). Tests of whether all study-specific effects exceed a specified (non-zero) value are also available (Pan and Wolfe, 1997). Such tests would be useful as an assurance that an effect is truly generalizable across diverse scenarios (or studies). Unfortunately these tests also suffer from poor power in typical situations (Piantadosi and Gail, 1993).

### 3.6 Summarizing remarks

In the absence of a specific distributional assumption for *f*(Φ), we can address some, but not all, of our important objectives. Point estimation of *μ* and *τ*^2^ poses no problem, although confidence intervals rely on approximating distributions, sometimes based on the central limit theorem, which may not be realistic when there are few studies. Several statistical tests, and recent methods for quantifying the effect of heterogeneity, are available. The objective that we consider to be most important, however, that of prediction, cannot satisfactorily be addressed.

## 4 Classical parametric and Bayesian approaches to random-effects meta-analysis

On observing the limitations of distribution-free random-effects approaches, we are led to consider parametric alternatives. From a classical point of view, interpretation of parametric random-effects meta-analysis is formally in the context of an imaginary ‘universe’, or ‘population’ of similar effects from which the effects in the observed studies are independently and identically sampled. Such an infinite population does not in reality exist, although the construct does allow inference about treatment effects to be extended to a broader population than the studies at hand.

A common misinterpretation of the random-effects assumption is that the *studies themselves are sampled* from a population of studies (Hedges, 1987; Anello and Fleiss, 1995; Colditz *et al.*, 1995). This implies that the types of participants, natures of exposures, design aspects, and so on, are randomly selected from a distribution of possibilities, which is an inappropriate interpretation because studies are individually designed dependent on the results of previous studies (Peto, 1987) and the evolutionary process of scientific research (Colditz *et al.*, 1995). Random sampling of participants would correspond to an assumption of a random-effects distribution for baseline parameters (such as baseline risks or means). Several researchers have implemented such an assumption, although we consider it an unnecessary constraint that we prefer to avoid.

Fundamental to a Bayesian approach to random-effects meta-analysis is the concept of *exchangeability*. Exchangeability represents a judgement that the treatment effects may be non-identical but their magnitudes cannot be differentiated *a priori* (Skene and Wakefield, 1990; Gelman *et al.*, 1995). Formally, we say that the joint distribution of underlying effects *p*(*θ*_1_,…,*θ*_*k*_) is identical under any permutation Π of the subscripts (Bernardo and Smith, 1994): 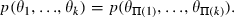


Exchangeability describes the *a priori* position of expecting underlying effects to be similar, yet non-identical. It also reflects a degree of prior ignorance in that the magnitudes of the effects cannot be differentiated. If particular covariates are believed to be important then an exchangeability assumption would not be appropriate. However, partial exchangeability, i.e. exchangeability for the residual effects conditional on the effect of the covariates, may be a reasonable position.

If the underlying effects are deemed to be exchangeable then, under broad conditions, it follows that the *θ*_*i*_ are independent and identically distributed conditional on *f*(Φ) (Bernardo and Smith, 1994; O'Hagan, 1994). The form of the distribution is a further judgement of the meta-analyst. Since exchangeability, and hence the random-effects distribution for the treatment effects, is a judgement of similarity, the Bayesian approach requires no sampling mechanism for the generation of the *θ*_*i*_. We see this as a substantial philosophical advantage over the frequentist approach.

A simple hierarchical Bayesian model, assuming exchangeability, for meta-analysis of estimates 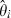 with variances 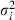 is 
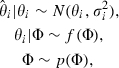
 where *p*(Φ) is some joint prior distribution. The choice of prior distribution, even among those that are intended to be non-informative, can have substantial effects on the results of the analysis (Lambert *et al.*, 2005). We implement our Bayesian analyses by using Markov chain Monte Carlo (MCMC) methods, with the software WinBUGS (Spiegelhalter *et al.*, 2003). All inferences can be readily accompanied by a relevant statement of uncertainty. It is standard to assume prior independence of *μ* and *τ*^2^. Minimally informative (e.g. locally uniform) distributions are appropriate in the absence of good reason to introduce prior information. Example WinBUGS code appears in Appendix A. Other methods for performing Bayesian random-effects analyses are available (Pauler and Wakefield, 2000; Abrams and Sanso, 1998; DuMouchel, 1994).

## 5 Meeting the important objectives with a normal assumption for the random effects

No model for the random-effects distribution will be ‘true’, and we might seek a parsimonious model that adequately fits the observed data. Requiring only two parameters for its full specification, the most parsimonious choice is also the conventional choice, where *f*(Φ) is chosen to be a normal distribution: 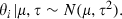
(10)

Standard random-effects meta-analysis methods make this assumption (Whitehead, 2002), and we now discuss the extent to which classical and Bayesian approaches with a normal distribution for the random effects allow the important questions to be addressed.

### 5.1 Heterogeneity

Several estimators of the heterogeneity variance *τ*^2^ are available that are based on normal likelihoods, including iterative maximum likelihood and restricted maximum likelihood estimates (DerSimonian and Laird, 1986; DerSimonian and Kacker, 2007). Some methods allow quantification of uncertainty in the estimate, including a full likelihood approach to the meta-analysis (Hardy and Thompson, 1996), and a method based on a *χ*^2^-distribution for a random-effects version of the *Q*-statistic (Viechtbauer, 2007; Knapp *et al.*, 2006); Viechtbauer (2007) reviewed several methods. Confidence intervals for measures of inconsistency can be calculated (Higgins and Thompson, 2002).

In a Bayesian analysis, a posterior distribution for *τ*^2^ is readily available. This is likely to be skewed, and the median and appropriate percentiles are typically used for point and interval estimation. Alternatively, Bayesian versions of generic approaches to quantifying the effect of heterogeneity may be used (Higgins and Thompson, 2002).

### 5.2 Mean effect

With the additional assumption of normality for the random effects, the confidence interval for the random-effects mean in expression (8) relies only on the assumption that *τ*^2^ (in addition to the 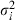) is estimated without uncertainty. To account for uncertainty in *τ*^2^, a *t*-distribution should provide a better basis than a normal distribution. An effective number of degrees of freedom for such a *t*-distribution is difficult to determine, since it depends on the extent of the heterogeneity and the sizes of the within-study standard errors as well as the number of studies in the meta-analysis. Several researchers have investigated the distribution of the Wald statistic 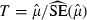, with suggestions to use *t*_*k*−4_ (Berkey *et al.*, 1995), *t*_*k*−2_ (Raghunathan and Ii, 1993) or *t*_*k*−1_ (Follmann and Proschan, 1999), or to use a modified Wald statistic with a *t*_*k*−1_-distribution (Hartung and Knapp, 2001a, b). Detailed discussion of the problem is available in Larholt (1989).

We make use of a compromise of *t*_*k*−2_, which allows application of methods for as few as three studies, yet also reduces the degrees of freedom compared with, say, the *k*−1 that are available when estimating the mean from a sample of observations (rather than the unobserved *θ*_*i*_ here). Better, likelihood-based approaches may be used that incorporate uncertainty in the estimation of *τ*^2^ (Hardy and Thompson, 1996; Vangel and Rukhin, 1999).

The posterior distribution for the underlying mean *μ* is readily available from a Bayesian meta-analysis. The posterior median and appropriate percentiles enable point and interval estimation.

### 5.3 Study effects

Empirical Bayes methods arise quite naturally from the classical normal random-effects model. Under the model that is defined by distributions (6) and (10), Bayes theorem determines that the conditional distribution of *θ*_*i*_ is given by 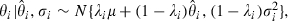 with 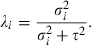


Substituting standard estimates for the unknown quantities yields a simple, parametric, empirical Bayes estimate of the effect in the *i*th study: 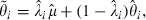 with approximate standard error 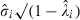, where 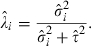


These expressions can be improved on to account for uncertainty in 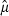 and 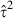 (Louis, 1991; Morris and Normand, 1992). The problem was tackled in particular by Morris (1983). Meta-analytic implementations have made use of the EM algorithm and bootstrapping (Raudenbush and Bryk, 1985; Laird and Louis, 1989).

The posterior distributions for the study-specific effects *θ*_*i*_ are readily available from a Bayesian analysis. These provide fully Bayesian shrunken estimates, in which inference for each particular study is performed by ‘borrowing strength’ from the other studies.

### 5.4 Prediction

The derivation of the predictive distributions for the effect *θ*_new_ in a future study has received little methodological attention in a frequentist context. If *τ*^2^ were known, then 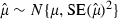 and *θ*_new_∼*N*(*μ*,*τ*^2^) imply (assuming independence of *θ*_new_ and 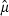, given *μ*) that 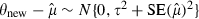 and hence that 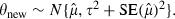


A realistic predictive distribution must recognize, however, that *τ*^2^ is estimated, usually from a small number of studies, and imprecision in 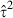 affects both terms in the variance of *θ*_new_. Again taking a *t*-distribution with *k*−2 degrees of freedom, we assume that, approximately, 
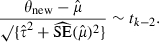
(11)

Thus an approximate 100(1−*α*)% prediction interval for the effect in an unspecified study can be obtained as 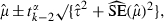
(12) where 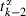 is the 100(1−*α*/2)% percentile of the *t*-distribution with *k*−2 degrees of freedom.

Within a Bayesian framework using MCMC methods, a predictive distribution for the effect in a new study is available by sampling such a new study, *θ*_new_∼*N*(*μ*,*τ*^2^) (Smith *et al.*, 1995). A 95% prediction interval for the new study may be obtained from the 2.5% and 97.5% quantiles of the posterior distribution of *θ*_new_. Alternatively, an approach that illustrates uncertainty in the whole distribution is to exploit the probability density function of *θ*: 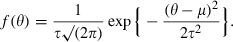


The value of *f* may be sampled in an MCMC analysis for a series of fixed values of *θ*. A smoothed plot of these produces an illustration of the predictive distribution. We prefer to plot the cumulative distribution function of the random-effects distribution as it facilitates derivation of useful posterior probabilities, as we discuss in Section 5.5. Computer code is provided in Appendix A, and an example is given in Section 7.

### 5.5 Testing

In the absence of a distributional assumption for the random effect, we saw that a test for qualitative interaction was available. With the addition of a normal distribution assumption, a classical hypothesis test of whether the mean of the random-effects distribution is non-zero is available. This may be performed as a Wald test based on 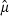, analogously to the test for the common effect. Equivalently, the test refers 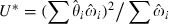
(13) to a 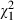-distribution. An improvement to this test, allowing for imprecision in 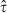, has been proposed by Hartung and Knapp (2001a, b). An approximate approach, which is based on distribution (11), may be used to test specific hypotheses about the predicted true effect in a new study, *θ*_new_.

Bayes factors are a Bayesian analogue of classical hypothesis tests and have been proposed in meta-analysis for testing for heterogeneity (Pauler and Wakefield, 2000) and for testing whether non-zero effects exist in specific studies (Kass and Raftery, 1995) and overall (Goodman, 1999). A particular advantage is that they may be used to provide evidence specifically in favour of a null hypothesis. However, it is unclear how prior distributions should be specified, which is an important limitation since Bayes factors can be very sensitive to them (Kass and Raftery, 1995). The deviance information criterion provides an alternative criterion, which is analogous to Akaike's criterion, which does not require proper prior distributions (Spiegelhalter *et al.*, 2002).

An alternative to hypothesis testing is to determine probabilities, based on the posterior distribution, for parameters being in particular regions. These are particularly useful for directly addressing specific, clinically relevant effects. Posterior probabilities for the underlying mean, such as *P*(*μ* <*μ*_0_) for some specified *μ*_0_, may be estimated as the proportion of MCMC iterations in which *μ* <*μ*_0_. For example, the probability that the mean effect is less than 0 provides a useful one-sided version of the test that is based on equation (13).

**Fig. 3:**
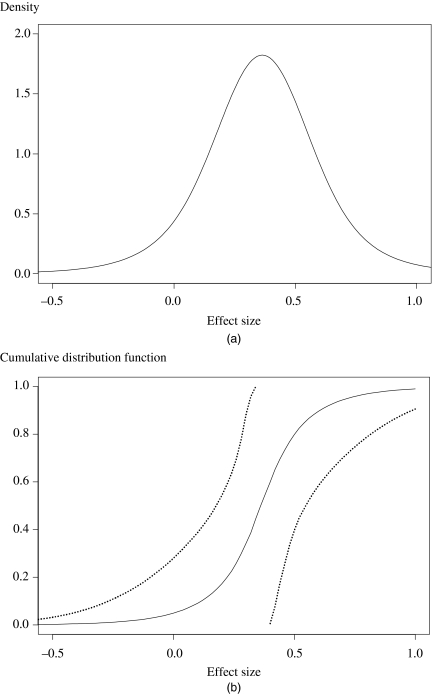
(a) Predictive distribution for *θ*_new_ and (b) cumulative distribution function of the random-effects distribution (with 95% interval) estimated from a Bayesian normal random-effects meta-analysis of set shifting studies

Probabilities for study effects may be obtained similarly by counting the proportion of MCMC iterations in which *θ*_new_<*θ*_0_. The proportion of positive (or negative) effect sizes provides an alternative to the test of qualitative interaction. Note that reading vertically from the cumulative distribution function in Fig. 3(b) in Section 7 directly provides estimates and 95% credibility intervals for *P*(*θ*_new_<*θ*_0_) for various values of *θ*_0_. Bayesian probabilities can address a variety of questions more readily than classical hypothesis tests.

### 5.6 Summarizing remarks

The assumption of a normal distribution for the random-effects distribution allows the calculation of confidence intervals and prediction intervals. Here we proposed approximate prediction intervals that incorporate uncertainty in both the mean effect and the heterogeneity parameter. The true predictive distribution is a complex function of the degree of heterogeneity, the number of studies and the within-study standard errors. An advantage of a Bayesian approach to random-effects meta-analysis over a classical implementation of the same model is the allowance for all uncertainties, particularly in obtaining a predictive distribution for the true effect in a new study. This may be important when there are few studies in the meta-analysis. However, this uncertainty does not extend to the choice of model for the random effects, which is more difficult to defend empirically when there are few studies. Bayesian posterior probabilities in particular can depend heavily on the distribution of the assumed likelihood and prior specification. A second potential advantage of a Bayesian approach is the intuitive interpretation of posterior distributions in terms of probability, including the ready availability of probabilities that effects exceed specified levels.

## 6 Non-normal and flexible assumptions for the random effects

Using a normal distribution for the underlying effects in different studies is a strong assumption, which is usually made in the absence of convincing evidence in its favour. The particular form that is assumed for the distribution can impact on the conclusions of the meta-analysis. Alternative parametric distributions have been suggested for *f*(Φ). For example, a *t*-distribution or skewed distribution might be used to reduce the effect of outlying studies (Smith *et al.*, 1995; Lee and Thompson, 2007), and mixture distributions have been advocated to account for studies belonging to unknown groupings (Böhning, 2000). Using MCMC methods in a Bayesian framework, parametric distributions for the random effects offer the same opportunities for inference as a normal distribution. However, some parametric distributions may not have parameters that naturally describe an overall mean, or the heterogeneity across studies. In a classical framework, the major limitation in applying non-normal parametric random-effects distributions is the computational complexity in performing the inference (Aitkin, 1999a).

Meta-analyses of a large number of large studies lend themselves to relatively complex models, since there may be strong evidence against a normal distribution assumption for the random effect. At the extreme, models may be fitted that are so flexible that the observed data determine the shape of the random-effects distribution. Below we discuss two examples of these flexible approaches that have received particular attention: in a classical framework, non-parametric maximum likelihood (NPML) procedures and, in a Bayesian framework, semiparametric random-effects distributions.

NPML procedures provide a discrete distribution that is based on a finite number of mass points (Laird, 1978; Böhning, 2005). Estimation can be achieved via the EM algorithm, and the number of mass points, along with compatibility of the data with different parametric or common effect models, can be determined by comparing deviances (Aitkin, 1999a, b). Estimates of the overall mean and the heterogeneity variance *τ*^2^ are available (Van Houwelingen *et al.*, 1993; Aitkin, 1999b), as are empirical Bayes estimates for the individual studies (Stijnen and Van Houwelingen, 1990). A particular advantage of the NPML approach is its ability to detect and incorporate outliers. However, NPML has been noted to be unstable (Van Houwelingen *et al.*, 1993) and has not been widely adopted. Furthermore, the key difficulty with NPML with regard to our important objectives is that realistic predictions are unlikely to follow from the discrete distribution for the random effects, especially when the studies include one or more outliers.

Bayesian semiparametric random-effects distributions have been proposed (Burr *et al.*, 2003; Burr and Doss, 2005; Ohlssen *et al.*, 2007) based on Dirichlet process priors. These comprise discrete mass points that are drawn from a baseline distribution, which might be normal, weighted in such a way that a single parameter *α* controls how close the discrete distribution is to the baseline: high values of *α* correspond to a random-effects distribution that is close to the baseline; low *α* to an arbitrary shape. We can in principle use the observed data to learn about *α*, although the small or moderate number of studies in a typical meta-analysis would carry little information. A truncated Dirichlet process offers a more convenient way to implement these models, in which a specified number of mass points is assumed (Ohlssen *et al.*, 2007), and a mixture of Dirichlet processes allows the discrete points to be replaced by normal distributions, so that the fitted random-effects distribution is a continuous, highly adaptable, mixture of normal distributions. These models can be fitted in WinBUGS, offering a variety of statistical summaries of the posterior distribution for the random effect: nevertheless it must be acknowledged that the analysis becomes considerably more complex. In common with classical non-parametric methods, predictions arising from Dirichlet process priors may be unhelpful, as they can have unconventional shapes that depend heavily on the particular studies that are included in the meta-analysis, although stronger assumptions about *α* can constrain the distribution to be reasonably ‘close’ to a baseline distribution with a specified shape.

## 7 Example

We take an example of 14 studies comparing ‘set shifting’ ability (the ability to move back and forth between different tasks) in people with eating disorders compared with healthy controls to illustrate the flexibility of a Bayesian approach to random-effects meta-analysis. Effect sizes, calculated as standardized differences in means, taken from a systematic review of the topic are reproduced in Table 1 (Roberts *et al.*, 2007). These are based on differences in set shifting ability using the ‘trail making’ task; positive effect sizes indicate greater deficiency in people with eating disorders.

**Table 1:** Summary data from 14 comparative studies of set shifting ability in people with disorders and healthy controls (Roberts *et al.*, 2007) with a classical meta-analysis using an inverse variance weighted average with random effects based on estimated standardized mean differences and a moment estimate of between-study variance (Whitehead and Whitehead, 1991)†

*Study*	*Effect size (standardized difference in means)*	*Standard error of effect size*
Steinglass	0.38	0.40
Holliday	0.07	0.21
Tchanturia	0.52	0.29
Tchanturia, study 1	0.85	0.25
Tchanturia, study 2	0.45	0.29
Murphy, study 1	0.01	0.35
Murphy, study 2	−0.58	0.36
Mathias and Kent	0.44	0.25
Kingston	0.46	0.22
Thompson	0.93	0.47
Jones, study 1	0.28	0.24
Jones, study 2	0.20	0.28
Jones, study 3	0.46	0.23
Witt	0.59	0.36

† Test for heterogeneity (equation (7)): *Q* =16.73, 13 degrees of freedom (*P* =0.02); *I*^2^=22%. Random-effects meta-analysis: standardized mean difference 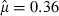 (95% credible interval 0.19–0.53); U^*^=4.23 (*P* =2×10^−5^); 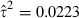.

**Fig. 2:**
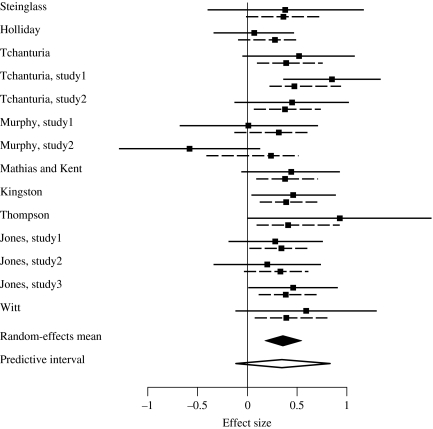
Bayesian normal random-effects meta-analysis of the set shifting data: for each study the estimated effect size with 95% confidence interval (Table 1) and a posterior median with 95% credible interval are illustrated; 95% credible intervals for *μ* and for the predicted effect in a new trial, *θ*_new_, are given

Classical normal random-effects meta-analysis results as traditionally presented appear at the bottom of Table 1. There is some heterogeneity between the effect sizes, accounting for 22% of the variation in point estimates by using the statistic *I*^2^ (Higgins and Thompson, 2002). The estimate of the heterogeneity variance is 0.022 and the test for heterogeneity is statistically significant at conventional levels of significance. It is evident from Table 1, as well as from the estimated effect sizes, plotted with 95% confidence intervals in Fig. 2, that the majority of trials indicate greater set shifting deficiency in people with eating disorders. Correspondingly, the test for qualitative interaction (9) produces a statistically non-significant test statistic Q^*^=2.56, compared with a critical value of 14.15 for a significance level of 5% (Gail and Simon, 1985). A random-effects meta-analysis reveals a statistically significant benefit *on average*, based on the inference in equation (13) regarding *μ* alone. The approximate prediction interval (12) for the true effect in a new study, however, ranges from −0.01 to 0.74, which is slightly less convincing.

Results from a Bayesian normal random-effects meta-analysis appear in Table 2 and are illustrated in Figs 2 and 3 (the computer code is in Appendix A). We used a uniform prior distribution for *τ* (>0) and a vague *N*(0,10^3^) prior distribution for *μ*. Results for *μ* and *θ*_new_ are comparable with the classical results, with credible intervals being wider because the Bayesian analyses properly account for uncertainty in the estimation of *τ*^2^. Fig. 3 shows the predictive distribution of *θ*_new_ and the cumulative distribution function for the random-effects distribution (with associated uncertainty). Note that *P*(*θ*_new_<0)=0.950 in Table 2 corresponds to the point at which the full curve in Fig. 3(b) crosses the vertical axis (at approximately 5% probability), and a credible interval around this probability is available from the dotted curves in Fig. 3(b).

**Table 2:** Summaries from posterior distributions after Bayesian normal random-effects meta-analysis of set shifting data by assuming a normal distribution for the random effects

*Parameter*	*Median*	*95% credible interval*	*P(parameter > 0)*
*μ*	0.36	(0.18, 0.55)	0.999
*θ*_new_	0.36	(−0.12, 0.84)	0.950
*τ*^2^	0.023	(0.000024, 0.196)	—

Various sensitivity analyses have small effects on the results, though they do not materially affect the conclusions from the Bayesian meta-analysis. Using a *t*-distribution with 4 degrees of freedom for the random effects yields a prediction interval from −0.16 to 0.89 (*P*(*θ*_new_<0)=0.947). Alternatively using an inverse gamma distribution for *τ*^2^ with parameters 0.001 and 0.001 (Smith *et al.*, 1995) produces a prediction interval from −0.008 to 0.73 (*P*(*θ*_new_<0)=0.973), and using the shrinkage prior that was advocated by DuMouchel and Normand (2000) produces a prediction interval from −0.009 to 0.72 (*P*(*θ*_new_<0)=0.973). The data in this example were available only as effect sizes and their confidence intervals, from which we calculated standard errors. For some applications, more detailed data are available and should be explicitly modelled. For example, when 2×2 contingency tables are available for binary outcomes, meta-analyses are appropriately performed by using binomial likelihoods (Smith *et al.*, 1995; Warn *et al.*, 2002).

## 8 Some special considerations in random-effects meta-analysis

### 8.1 Diversity and bias

An important distinction to make when considering heterogeneity of effect sizes is between the effects of diversity (variation in populations, interventions, exposures, outcome measures etc.) and bias (variation in the quality of the design and conduct of the studies) (Glasziou and Sanders, 2002; Greenland and O'Rourke, 2001). Suppose that we represent the true effect in the absence of biases in the *i*th study by *Φ*_*i*_. Variation in *Φ*_*i*_-parameters reflects diversity, and we represent this by using a normal distribution 
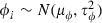
 across the ‘true’ (unbiased) effects. Let us summarize the biases at the study level as *β*_*i*_. The effect *being evaluated* in the *i*th study, *θ*_*i*_, is the sum of the true effect and bias: *θ*_*i*_=*Φ*_*i*_+*β*_*i*_. The important point is that the desired inference concerns the distribution of true effects only. We wish to estimate *μ*_*Φ*_, and 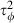, and possibly each *Φ*_*i*_, and to make inferences on the predictive distribution 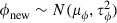.

If study level covariates are available that summarize the risk of bias in a study, then these may be incorporated in a random-effects metaregression analysis. Providing that these are centred at a value indicating freedom from bias, then the intercept and residual heterogeneity from this analysis suitably estimate *μ*_*Φ*_ and 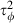 respectively. Quality scores or a series of covariates targeting specific sources of bias may be used. There are strong arguments against the former (Greenland and O'Rourke, 2001) and an item-based approach to dealing with quality is to be preferred (Jüni *et al.*, 2001). Such an analysis may not always be possible or appropriate. Design and conduct are difficult to assess, particularly from reports of studies, and important information may be unavailable. Sometimes there is no clear ‘best’ category. Sources of control groups, degrees of adjustment for confounders and use of different outcome measures or laboratory techniques provide some examples of this. Furthermore, if there are no studies falling into the ‘best’ category for any item, then the intercept in the metaregression analysis cannot be estimated.

The following approach, which was described by Spiegelhalter and Best (2003), might help to separate heterogeneity due to biases from heterogeneity due to diversity. Suppose that the study-specific bias components follow a normal distribution such that 
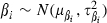
. The random-effects model may then be written as follows, assuming that the diversity and bias components are uncorrelated: 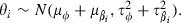


This may be written as 
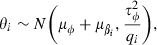
 with ‘quality weights’ of the form 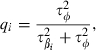 describing the proportion of between-study variability due to true variability rather than bias: these can be either fully specified or have informative prior distributions placed on them. Specification of *μ*_*β*_*i*__ and 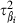 can become arbitrarily complex. Greenland and O'Rourke (2001) pointed out that *μ*_*β*_*i*__ might be separated into components that are observed (particular aspects of the studies that are known) and bias components that are unobserved. Alternative approaches to dealing with specific sources of bias are available (Eddy *et al.*, 1992; Greenland, 2005). The usual random-effects model (10) makes a strong assumption that the distributions of the study-specific biases *β*_*i*_ are identical across studies, thus giving *μ* =*μ*_*Φ*_+*μ*_*β*_ and 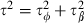. Indeed, Cox (2006) remarked that

‘an assumption that systematic errors in different sections of the data are independent random variables of zero mean deserves extreme caution’.

In practice, it can be difficult to separate diversity from bias, and it is partly this lack of identifiability that often necessitates the use of the conventional random-effects model. In the presence of potential biases, therefore, results from a random-effects model are challenging to interpret. The parameters underlying the bias distributions could also, in principle, be either subjectively assessed (Turner *et al.*, 2008) or estimated by using analyses of multiple meta-analyses (Welton *et al.*, 2008).

### 8.2 Reporting biases and small study effects

A covariate that may be influential and is highly relevant to random-effects meta-analyses is study size. A relationship between treatment effect and study size (or study precision) induces an asymmetric funnel plot. This may reflect publication bias, or correlation between study size and another important covariate such as quality of study (Egger *et al.*, 1997). Whatever the reason for it, an asymmetric funnel plot leads necessarily to a difference in point estimates between common effect and random-effects meta-analyses (using standard inverse variance methods), because random-effects methods give relatively more weight to less precise studies. Although this discrepancy is not of direct concern to us here, particular caution is warranted when interpreting any meta-analysis when a relationship exists between treatment effect and study size.

### 8.3 Small numbers of studies

Meta-analyses with very small numbers of studies (say 2–4) pose particular problems in a classical framework since the estimation of *τ*^2^, and hence of *μ*, is highly imprecise. It may be tempting to resort to a common effect model, often superficially backed up by the lack of a statistically significant test for heterogeneity. Tests for heterogeneity have low power when there are few studies, and heterogeneity may be just as likely for small numbers of studies as it is for large numbers of studies. Thus the rationale is not strong for opting for a common effect model in this situation. It is usually worth considering whether presentation of the results of individual studies is more informative than a meta-analysis (Cox, 2006). If a meta-analysis is to be undertaken, then plausible values for *τ*^2^ may be preferable to values that are estimated from very few studies. A natural alternative is to take a Bayesian approach and to inform the analysis by using external information, or prior beliefs. For example, an informative prior distribution for *τ*^2^, or equivalently on *τ*, can be used, based on observed heterogeneity variances in other, similar, meta-analyses (Higgins and Whitehead, 1996). It has been suggested that a prior distribution on *τ* might comprise the positive part of a symmetric distribution around 0, say Cauchy or normal (Gelman, 2006).

## 9 Discussion

We have re-evaluated the role of meta-analysis in the presence of heterogeneity and noted some limitations of commonly used methods. In particular,

- implementing a common effect model produces confidence intervals that do not allow for variation in underlying effects and should be avoided
- testing for heterogeneity addresses an unimportant question (it is preferable to measure the extent of heterogeneity) and
- focusing on the mean of a random-effects distribution is insufficient.

It may be a common misconception that the confidence interval for the underlying mean in a random-effects meta-analysis, because it is typically wider than for a common effect meta-analysis, incorporates a measure of the extent of heterogeneity. However, as we demonstrate in Fig. 1 this is not so, and appropriate consideration must be given to the whole distribution to avoid misleading generalizations about effects across studies. We argue that predictive distributions are often the most sensible way to summarize the results of heterogeneous studies, and we propose a simple method for producing a prediction interval within a classical framework. These considerations apply to numerous other applications of random-effects methods.

We have, for the most part, concentrated on the usual normal distribution assumption for random effects. In many situations this is the most feasible assumption given the limited number of studies and the lack of strong evidence for or against the assumption. Flexible distributions such as Bayesian semiparametric models are available, although it remains difficult to implement non-normal distributions outside an MCMC approach to the analysis. Considerably more detailed modelling of heterogeneity, particularly that due to biases, can be performed when sufficient information is available.

We note that there are situations in which random-effects meta-analyses should be used with great caution, or not at all, e.g. when there is important funnel plot asymmetry, or if there is substantial variation in the direction of effect or if there are obvious covariates that should be modelled by using a metaregression. Furthermore, certain common effect approaches have practical advantages over random-effects methods. For example, they are easier to implement, and the one-step ‘Peto’ method has been observed to have desirable properties when binary events are rare (Bradburn *et al.*, 2006).

In conclusion, we offer some recommendations for practice. For meta-analyses of a moderate number of studies, we recommend that

- visual inspection of a plot, such as a forest plot, is used as a preliminary inspection of heterogeneity
- heterogeneity is allowed for in a meta-analysis using covariates and/or random effects
- random-effects meta-analyses are interpreted with due consideration of the whole distribution of effects, ideally by presenting a prediction interval, and
- statistical tests focus on the important questions of whether an effect exists anywhere and whether it has a consistent direction across studies.

All these can be achieved by using a classical framework; we propose a simple prediction interval in expression (12). A Bayesian approach has the additional advantages of flexibility, allowing incorporation of full uncertainty in all parameters (but not uncertainty in the model) and of yielding more readily interpretable inferences. If a normal distribution for the random effects cannot be assumed then non-parametric approaches (such as Bayesian semiparametric prior distributions) should be considered. For meta-analyses involving substantial numbers of studies, covariates representing sources of diversity and bias should be considered, and the latter should ideally be excluded from prediction intervals. Meta-analyses of very few studies need not ‘default’ to a common effect model, since informative prior distributions for the extent of heterogeneity can be incorporated in a simple Bayesian approach.

## Appendix A

WinBUGS code is shown for random-effects meta-analysis of estimates *y* and variances *v*. Other data required are *k* (the number of studies) and low and high (a range for plotting the probability density function, e.g. from −1 to 1).
